# CuO Nanoparticle-Mediated Seed Priming Improves Physio-Biochemical and Enzymatic Activities of *Brassica juncea*

**DOI:** 10.3390/plants12040803

**Published:** 2023-02-10

**Authors:** Ahmad Faraz, Mohammad Faizan, Vishnu D. Rajput, Tatiana Minkina, Shamsul Hayat, Mohammad Faisal, Abdulrahman A. Alatar, Eslam M. Abdel-Salam

**Affiliations:** 1 Department of Biotechnology, School of Life Sciences, Glocal University, Saharanpur 247121, India; 2 Botany Section, School of Sciences, Maulana Azad National Urdu University, Hyderabad 500032, India; 3 Academy of Biology and Biotechnology, Southern Federal University, 344006 Rostov-on-Don, Russia; 4 Plant Physiology Section, Department of Botany, Aligarh Muslim University, Aligarh 202002, India; 5 Department of Botany & Microbiology, College of Science, King Saud University, P.O. Box 2455, Riyadh 11451, Saudi Arabia; 6 Plant Molecular Biology, Faculty of Biology, Ludwig-Maximilians-University Munich, 82152 Planegg, Germany

**Keywords:** *Brassica juncea*, copper oxide, nanoparticles, photosynthetic rate, proline, antioxidant activities

## Abstract

The use of nanoparticles (NPs) in agricultural fields has risen to a level where people are considering NPs as an alternative to commercial fertilizers. The input of copper oxide NPs (CuO NPs) as seed primers was investigated in this study, and the growth indices of *Brassica juncea* such as phenotypic parameters, photosynthetic attributes, and biochemical parameters were measured during maximum vegetative growth stage, i.e., at 45 days after sowing. Surface sterilized seeds were soaked in varying concentrations (0, 2, 4, 8 and 16 mg/L) of CuO NPs for 15, 30, and/or 45 min. After those priming periods, the seeds were planted in pots and allowed to grow naturally. Among the different tested concentrations of CuO NPs, 4 mg/L of CuO NPs for 30 min seed priming proved to be best, and considerably increased the, shoot length (30%), root length (27%), net photosynthetic rate (30%), internal CO_2_ concentration (28%), and proline content (41%). Besides, the performance of the antioxidant enzymes, viz, superoxide dismutase, catalase, peroxidase, and biochemical parameters such as nitrate reductase and carbonic anhydrase were also increased by several folds after the application of CuO NPs in *B. juncea*. The present study suggests that CuO NPs can be effectively used to increase the performance of *B. juncea* and may also be suitable for testing on other crop species.

## 1. Introduction

Nanoparticles (NPs) are tiny particles having at least one dimension between 1–100 nanometers, which are distinguished by their small size, which results in a high surface area-to-volume ratio and higher reactivity when compared to bigger particles or metals of similar composition. Scientists are now exploring the application of NPs in different areas such as biomedical, agricultural, and environmental fields [1]. Currently, more than thousands of commercially available products contain various types of NPs [2,3]. Research is underway to develop NPs that can improve the nutrient and pesticide delivery to plants [4]. NPs are used in agriculture as nano-fertilizers to increase plant growth, as pesticides to manage the harmful effects caused by pests on the crop, and sometimes also as sensors to monitor the soil quality and plant health [5]. The extensive use of NPs in various fields raises the concern that they are released into the environment and interact with the plant through their shoot part or root part positively or negatively [6,7]. Copper oxide nanoparticles (CuO NPs) have been reported to have a range of impacts on plants, which may have both positive and negative effects on plant growth and development. In several studies, beneficial effect of CuO NPs such as higher growth and production; greater stress tolerance in plants; a better defense mechanism against pathogens; and improved nutrient uptake and utilization in plants have been reported.

CuO NPs are nanometer-scale copper oxide particles that are a form of metal oxide NPs with distinctive physical and chemical features such as a large surface area, high reactivity, and a significant absorption capacity. CuO NPs have these properties, which make them appropriate for a wide range of applications in domains such as energy, electronics, and biology. Copper (Cu) is an important essential plant nutrient present in the soil, air, and/or water interacting with seeds or roots. The total worldwide Cu production amounted to an estimated 21 million metric tons in 2021. Global Cu production has seen steady growth over the past decades, rising from 16 million metric tons in 2010. CuO NPs are used extensively as catalysts, gas sensors, environmental remediation, solar cell, heat transfer fluids, drug delivery and in the manufacturing of semiconductors and photovoltaic cells [8,9]. They can be used in several different forms as described by Xiong et al. [10]. Especially, CuO NPs have been applied to control herbicides and fungicides. Some recent studies demonstrated that CuO NPs can be used as nano fertilizers [11,12]. *Cajanus cajan* treated with biogenic CuO NPs showed a positive response to growth indices as published by Shende et al. [13]. On the other hand, *B. nigra* germination seedling growth is inhibited when exposed to CuO NPs [14]. Adhikari et al. [15] investigated the role of CuO NPs in *Glycine max* and *Cicer arietinum* up to a level of 2000 mg/L and reported both positive and negative outcomes. It has been reported that at low concentrations of Cu NPs (5–20 mg/L), Cu accumulation and ROS generation occur, which generate metabolic effects. At 5 mg/L concentrations, CuO NPs added to *Arabidopsis thaliana* enhanced the flavonoid content [16]. Consequently, it is clear that CuO NPs may affect the plant life cycle in either a positive or negative way, depending on the quantities, structure, and concentration of NPs as well as the plant species used. The potential of CuO NPs for sustainable agriculture is enormous and global, but it needs more proteomic studies to conclude.

Several studies have been reported while investigating the role of NPs in seed priming, and very promising results were found. Improved growth performance in terms of germination and better photosynthetic attributes through seed priming was previously investigated in *Gossypium* by CeO_2_ NPs, *Triticum aestivum* by ZnO NPs, *Solanum lycopersicum* by AgNPs and TiO_2_ NPs, and *Zea mays* by ZnO NPs [17,18,19,20]. All of these studies show that NPs can be used effectively as seed primers to improve plant growth and performance.

*B. juncea* is a rabi crop that is mostly grown on irrigated land in India’s Indo-Gangetic plains. It is an annual herb with a maximum height of one meter. *B. juncea* is a key oil seed crop that is largely grown in arid and semi-arid areas [21]. In India, it is the second most significant crop for the production of edible oil, accounting for almost 27.8% of the Indian oilseed economy [22], with a global production of around 7% [23]. *B. juncea* is known to produce several bioactive phyto-chemicals including glycosides, flavonoids, phenolic compounds, sterol and triterpene alcohols, proteins, and carbohydrates.

Therefore, keeping these points in mind, the present research was conducted with an aim to explore the effect of CuO NPs through seed soaking on the performance of the *B. juncea*. The nano form of CuO significantly increases the plant growth and physiological and biochemical parameters. Antioxidant activity also enhances CuO NPs by seed soaking treatment. A number of studies have already been carried out with CuO NPs utilized for foliar spray application in different plant species. It is necessary that scientists try to investigate the effects of CuO NPs on plants at the beginning of their lifecycles, such as during seed germination.

## 2. Results

### 2.1. Phenotypic Character

**Figure 1:**
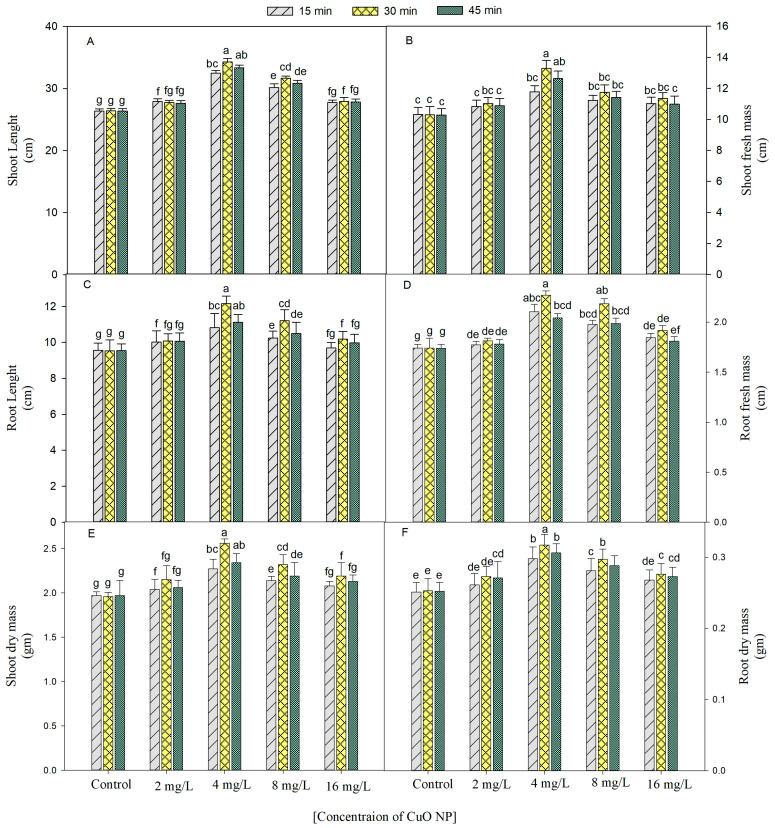
Outcome of CuO NPs on shoot (**A**) and root (**C**) lengths, fresh biomass of shoot (**B**) and root (**D**), and dry biomass of shoot (**E**) and root (**F**) of *Brassica juncea* at 45 DAS. All the data represent the mean of five replicates (*n* = 5); significant differences between control and treatment and among treatments were represented by different letters and standard error (±SE) was represented by vertical bars.

**Figure 2:**
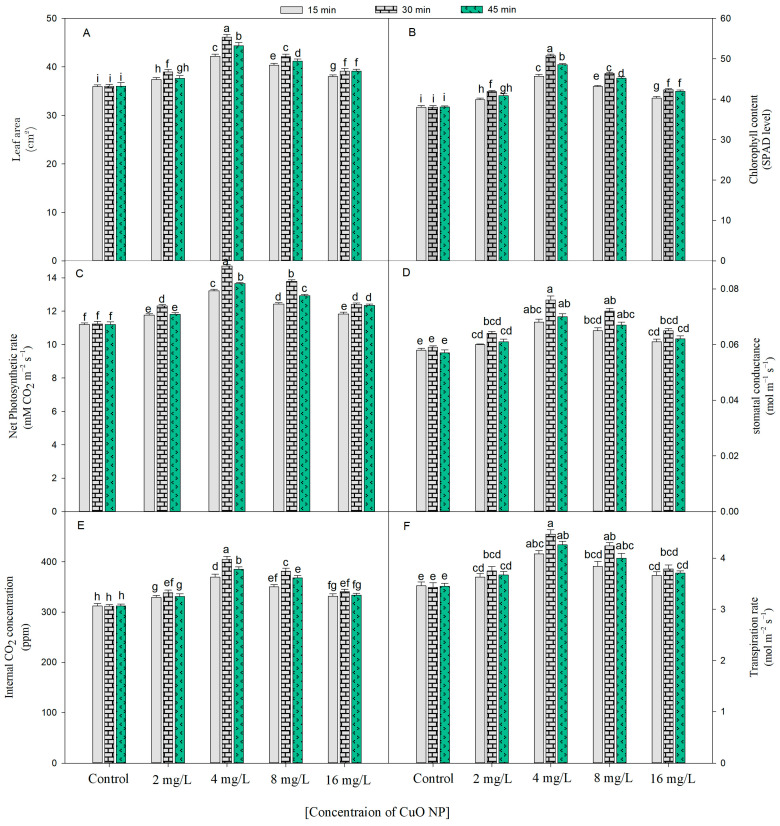
This figure depicts the outcome of CuO NPs on the leaf area (**A**) and SPAD value of chlorophyll (**B**), net photosynthetic rate (**C**) and stomatal conductance (**D**), internal CO_2_ concentration (**E**), and transpiration rate (**F**) of *Brassica juncea* at 45 DAS. The entire set of data shows the average of five replicates (*n* = 5); significant differences between control and treatment and among treatment were represented by different letters and standard error (±SE) was represented by vertical bars.

The growth (shoot and root length, fresh and dry weight, and leaf area) of *B. juncea* was increased by seed priming of CuO NPs (2, 4, 8, and 16 mg/L) for 15, 30, and 45 min at 45 days after sowing (DAS) (Figure 1A–F and Figure 2A). The optimal increase for all the growth characteristics was observed in the plants developed from the seeds treated with 4 mg/L of CuO NPs for 30 min over the untreated control plants, and the respective increase was 1.3 times (shoot and root length), 1.4 times (fresh weight), 1.3 times (dry weight), and 1.2 times (leaf area), at 45 DAS, over their respective controls.

### 2.2. Effect of CuO NPs on Physiological Indices

#### 2.2.1. Chlorophyll Content (SPAD Value)

As evident from Figure 2B, the SPAD values in the *B. juncea* plant increased by seed priming in CuO NPs prior to sowing and further increased as their growth progressed. The maximum SPAD value was recorded when the seeds were dipped in 4 mg/L of CuO NPs for 30 min and was about 1.3 folds more as compared with the control at 45 DAS.

#### 2.2.2. Photosynthetic Parameters

Photosynthetic attributes such as net photosynthesis (P*_N_*), stomatal conductance (gs), internal CO_2_ concentration (Ci), and transpiration rate (E) were substantially increased by the usage of CuO NPs through seeds. All the aforesaid parameters were increased irrespective of concentration and duration. However, the maximum increase in P*_N_*, g*_s_*, C*_i_*, and E by 1.25–1.35 folds was found after soaking the seeds in 4 mg/L of CuO NPs for 30 min as compared with their respective controls (Figure 2C–F).

### 2.3. Effects of CuO NPs on Biochemical Parameters

#### 2.3.1. Activity of Carbonic Anhydrase (CA) and Nitrate Reductase (NR)

**Figure 3:**
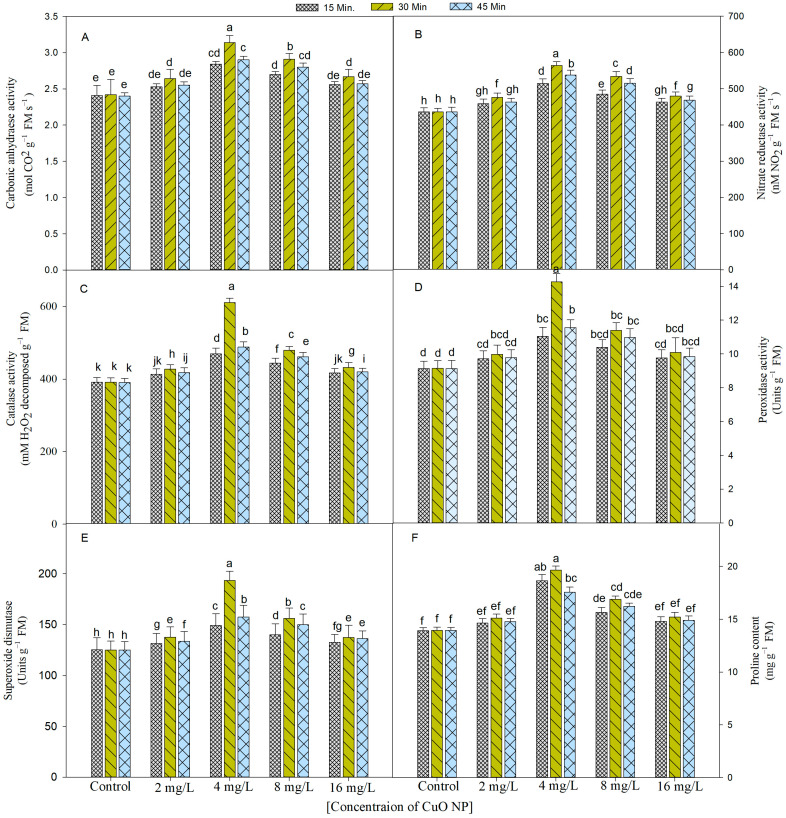
Graph shows the outcome of CuO NPs on the activity of carbonic anhydrase (**A**) and the activity of nitrate reductase (**B**), catalase (**C**), peroxidase (**D**), superoxide dismutase (**E**), and proline content (**F**) in *Brassica juncea* at 45 DAS. The entire set of data shows the average of five replicates (*n* = 5); significant differences between control and treatment and among treatments were represented by different letters and standard error (±SE) was represented by vertical bars.

The data presented in Figure 3A,B indicate that carbonic anhydrase (CA) and nitrate reductase (NR) activities of leaves were increased with the advancement of the age of the plant. They further increased with the treatment of CuO NPs. Maximum CA and NR activity were noted in the plants their seeds primed with 4 mg/L (30 min) of CuO NPs prior to sowing, which was 1.3 folds higher than the control treatments.

#### 2.3.2. Enzymatic Activity

The activity of antioxidant enzymes such catalase (CAT), peroxidase (POX), and superoxide dismutase (SOD) increased as the growth progressed and also in the plants that developed from the CuO NPs-treated seeds (Figure 3C–E). The maximum activity of these enzymes was recorded in the plants of the seeds dipped in 4 mg/L of CuO NPs for 30 min. The activity of CAT, POX, and SOD were increased by 56%, 55%, and 54%, respectively, over their respective controls. Other concentrations and durations of CuO NPs also increased the values for all the enzymes over their control.

#### 2.3.3. Proline Content

Plants raised with their seeds exposed to CuO NPs (2, 4, 8, and 16 mg/L) had significantly more proline content than the control irrespective of the duration of soaking (Figure 3F). Out of all these concentrations and durations, 4 mg/L of CuO NPs (30 min) proved the best and enhanced the proline content by 1.5 times as compared with their respective controls.

## 3. Discussion

The growth indices of *B. juncea* including the length of its shoots and roots and biomass (fresh as well as dry) were increased in the plants developed from the CuO NPs-treated seeds. This increase presumably may be because of the increased absorption of inorganic nutrients by the roots because of their better growth, which accelerated the breakdown of organic substances and led to increased growth performances [24]. Because of the smaller size and shape of NPs, the uptake of nutrients from the soil increases, leading to better surface area and increased photosynthesis, and finally enhanced the growth of the plants. Similarly, previous studies reported that NPs showed better growth performance in various crops such as carbon nanotubes (CNTs) in *B. juncea* [25], Zn, Fe, Cu NPs in *Vigna radiata* [26]*,* ZnO NPs in *C. arietinum* [27], Ag NPs in *T. aestivum* [28] and in *Trigonella foenum-graecum* [29], ZnO NPs and Fe_2_O_3_ NPs in *T. aestivum* [30], CuO NPs in *C. cajan* [31], and TiO_2_ NPs in *A. thaliana* [32].

Chlorophyll is proposed to be the backbone of photosynthesis and its increase in plants activates the process of photosynthesis [33]. In the present study, the seeds soaked in CuO NPs had increased chlorophyll (SPAD) values as compared with seeds soaked in distilled water. The maximum chlorophyll content was reported when the seed was soaked in 4 mg/L of CuO NPs for 30 min. This increase in the chlorophyll values may be attributed to the fact that CuO NPs may instigate translation and/or transcription of the enzymes involved in the biosynthesis of chlorophyll. Similarly, an increase in the level of chlorophyll by the use of other NPs has also been reported [34,35,36].

**Figure 4:**
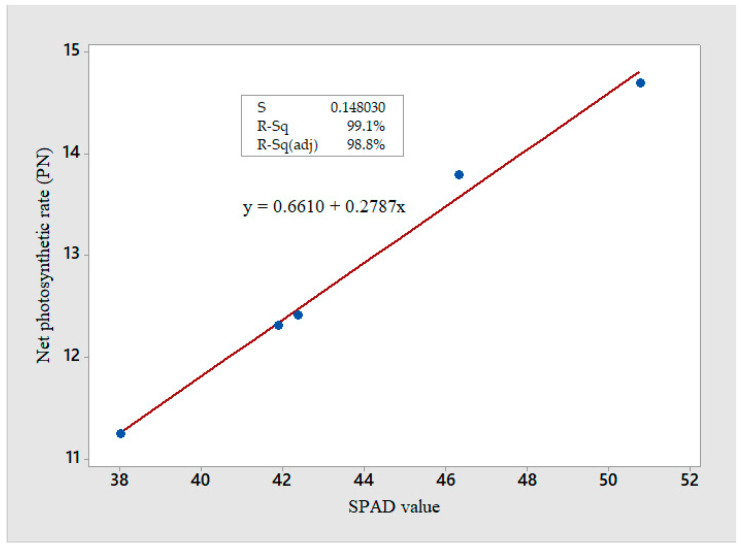
This graph shows the positive correlation between SAPD value and net photosynthetic rate which means that increases SPAD value has efficiently improved the photosynthetic rate.

Photosynthesis is one of the important indicators to envisage plant growth and productivity. It is considered one of the most vital processes in green plants. In our results, it was found that seeds soaking in CuO NPs before sowing increased the photosynthesis and related traits at all concentrations and duration. Similar results were also reported in earlier studies that NPs tend to alter the efficiency of photosynthesis in a positive or negative direction [37,38]. However, an increase in photosynthesis by TiO_2_ in *Spinacia oleracea* [39], SiO_2_ NPs in *Bambusa* [40], single-walled carbon nanotubes in *S. oleracea* [41], CuO NPs [42], and ZnO NPs in *S. lycopersicum* [35] is considered as a favorable impact. Moreover, an increase in photosynthetic activity with the stomatal movement has also been reported by Faizan et al. [43] with ZnO NPs in *S. lycopersicum* seedlings. Changes in gene expression and biosynthesis of specific proteins also alter the photosynthetic rate [44]. Xuming et al. [45] suggested that exposure of nano-anatase to *S. oleracea* leaves promoted the expression of small subunits (rbcs) and large subunits (rbcL) of Rubisco. Therefore, an increase in the amount of Rubisco mRNA and CA activity maximizes the carboxylation of Rubisco and P*_N_* [46]. A positive correlation between P*_N_* with SPAD values is seen in Figure 4, which further established that photosynthesis was regulated by multiple factors. Yang et al. [47] also reported a similar type of correlation between photosynthetic rate and chlorophyll content.

CA is a zinc-containing metalloenzyme that converts free atmospheric CO_2_ into HCO_3_ at the initial stages of photosynthetic reactions [48] and is also involved in various biological functions such as photosynthesis, ion exchange, respiration, and acid-base buffering [49]. Seed soaking treatment increased the CA activity, irrespective of the concentration of CuO NPs which influence the Rubisco activity which fixes CO_2_, thus improving the values for P*_N_*, g*_s_*, C*_i_*, and E. These results are well supported by recent research findings such as those of Siddiqui and Al-Whaibi [50], who observed that SiO_2_ NPs enhanced the rate of photosynthesis by altering CA activity in *S. lycopersicum* plants. Besides this, Siddiqui et al. [51], in *Cucurbita pepo* with SiO_2_ NPs; Ahmad et al. [52] in *Mentha × piperita* with TiO_2_ NPs; Faizan et al. [35] in *S. lycopersicum* with ZnO NPs; and Faraz et al. [36] in *B. juncea* with CuO NPs also reported a similar increase in CA activity.

Nitrate is the principal source of nitrogen to the plants acted upon by NR, the key enzyme which catalyzes the NAD (P)H-facilitated reduction into nitrite [53,54] to confirm the appropriate amount of nitrogen that should be present for the plant’s growth and development [55]. The activity of NR, in the present investigation, was increased by CuO NPs when the seeds were soaked before sowing (Figure 3B). The use of other types of NPs is also reported to improve the NR activity [52,56,57]. Plants treated with NPs showed high NR activity, and the reason behind this may be the stimulated gene expression which is involved in its synthesis because Das et al. [58] observed in the *Phaseolus vulgaris* that Ag NPs exposure improved the expression of genes related to NR synthesis. This increase in NR will naturally help the plants to reduce additional quantities of inorganic nitrogen to organic nitrogen to favor protein synthesis and growth.

In plants, ROS are not new things; they are produced in the form of by-products during the normal metabolic process of O_2_ and they play an important role in homeostasis and signaling [59]. Any irregularities in ROS activity may lead to oxidative stress, damage of DNA, protein, and lipids, and, lastly, result in the death of cells [60]. Plants have adopted to overpower these toxic effects by antioxidant enzymatic activity (CAT, POX, and SOD) and by nonenzymatic (proline) activity [61]; therefore, they are the key elements in the defense mechanism [62]. In the present study, *B. juncea* seed soaked in CuO NPs showed increased CAT, POX, and SOD levels. The reasons behind this increase in enzymes may be that NPs reach plant cells through root or leaf [63] and interfere with plant metabolism through nutrients [64], activate particular genes [65], and/or interfere with different oxidative processes [66]. Our results further corroborated the findings of others [13,35,36,67,68,69] who suggested that NPs have the potential to enhance the biosynthesis of antioxidant enzymes. In addition to this, Faizan et al. [70] found that ZnO NPs efficiently overcome the adverse effects caused by cadmium by increasing the antioxidant activity in rice plants, which improves the plant’s overall growth. Furthermore, NPs-induced SOD activity boosts plants’ capacities to cope with harmful oxide radicals, which would otherwise cause damage to the cell membranes [71]. Therefore, we can say that higher activity of these antioxidant enzymes (CAT, POX, and SOD) by CuO NPs application to *B. juncea* may be an alteration in the process of the central dogma system.

Proline, a highly soluble molecule with low molecular weight, provides a defense mechanism to plants against the stress through cellular osmotic adjustments to preserve membrane integrity and enzyme/protein stabilization [72,73]. Proline is the only molecule capable of protecting plants from highly harmful compounds that are produced when environmental conditions are unfavorable [74]. The outcome of the current study revealed that the application of CuO NPs as seed soaking enhanced the proline content of the leaves (Figure 3F). Increased proline content was also reported in mustard plant when treated with CuO NPs in the form of foliar spray [75]. The same result was also observed in cucumber by Zhao et al. [76]. Other NPs such as SiO_2_ NPs in squash [51]; TiO_2_ NPs in rice [77]; TiO_2_ NPs in barley [78]; ZnO NPs in tomato [79]; and CuO NPs in *Brassica* [36] also increased the proline content. All previous and present observations indicate that nanoparticles protect plants from stress by boosting their compatible solute, such as proline in *B. juncea*.

## 4. Materials and Methods

### 4.1. Nanoparticles and Seed Priming

The CuO NPs were procured from Sigma Aldrich Chemicals Pvt. Ltd. India, By dissolving the necessary amounts of CuO NPs in 10 mL of water in a 100 mL flask and adding double-distilled water (DDW) to a total volume of 100 mL, a stock solution of 16 mg/L CuO NPs was prepared. This stock solution was used to generate varied concentrations of CuO NPs (0, 2, 4, 8, and 16 mg/L) in which seeds of *B. juncea cv. RGN-48* were soaked. The Indian Agriculture Research Institute (PUSA, New Delhi, India), provided the seed of mustard *cv. RGN-48*.

### 4.2. Experimental Setup and Plant Growth Performance

For the experiment, uniform and healthy seeds were chosen. Before sowing, *B. juncea* seeds were soaked in varying concentrations (0, 2, 4, 8, and 16 mg/L) of CuO NPs for varying times (15, 30, and/or 45 min). Earthen pots containing manure and farm soil were used for the experiment. Each treatment had 5 replicates. The per pot received 50 mL water regularly. At 45 days stage, plants were harvested and randomly sampled to evaluate the various growth and physio-biochemical characteristics.

### 4.3. Determination of Phenotypic Characteristics

Plants were harvested after 45 days, uprooted with attached soil, washed with water to remove soil particles, and cut into shoot and root. Their length was measured using a meter scale. After recording the fresh weight, shoot and root were dried in an oven at 80 °C for 24 h to assess their dry weight.

### 4.4. Observation of Physiological Indices

#### 4.4.1. Leaf Area and Chlorophyll Content (SPAD Value)

An instrument leaf area meter (ADC Bioscientific, Hoddesdon, Herts, UK) was used to estimate the leaf area in fully expanded leaves. Chlorophyll content was measured as SPAD value and for this a Minolta chlorophyll meter (SPAD-502; Konica Minolta Sensing Inc., Tokyo, Japan) was used.

#### 4.4.2. Photosynthetic Rate and Their Related Parameters

A portable photosystem (model LI-COR 6400, LI-COR, Lincoln, NE, USA) was used to measure different photosynthetic parameters. With this instrument, we measured the values of P*_N_*, g*_s_*, E, and C*_i_* in the fully expanded leaves of plants. The air temperature, relative humidity, CO_2_ concentration, and Photosynthetic Photon Flux Density (PPFD) were maintained at 25 °C, 85%, 600 μmol mol^−1^, and 800 μmol mol^−2^ s^−1^, respectively.

### 4.5. Biochemical Parameters Estimation

#### 4.5.1. NR and CA Activity

The activity of NR was computed by the Jaworski [80] procedure. A mixture of newly form leaf (0.1 g), phosphate buffer (pH 7.5), KNO_3_, and isopropanol was stored in an incubator at 30 °C for 2 h. Sulfanilamide and N-1-napthylethylenediamine hydrochloride mixture were added to the incubated mixture. At 540 nm, the absorbance was read with a spectrophotometer (Spectronic 20D; Milton Roy, Ivyland, PA, USA). CA action in leaves was measured through Dwivedi and Randhawa [81]’s procedure. Leaves were slashed into minute pieces in a cysteine hydrochloride solution. They were blotted and conveyed in a test tube, phosphate buffer (pH = 6.8), 0.2 M NaHCO_3_, and bromothymol blue were added, and the red indicator of methyl. 0.5 N HCl was used for titrating.

#### 4.5.2. Estimation of Antioxidant Enzymes

For enzyme assay, fresh leaves were taken into account. All the necessary chemical and enzyme-extracting solutions were prepared by the methods given by Arya et al. with slight modification [82]. First, 1 g of fresh leaf samples was homogenized in enzyme extraction buffer and then the supernatant was collected, which was later used for the estimation of CAT, POX, and SOD activities using the method described by Faraz [83].

#### 4.5.3. Content of Proline

Bates et al. [84] method was used for the identification of the proline amount in newly formed leaves. Leaves extracted in sulfosalicylic acid and an equal volume of glacial acetic acid and ninhydrin solutions were added. The sample was heated at 100 °C, to which 5 mL of toluene was added. The absorbance of the aspired layer was read at 528 nm on a spectrophotometer.

### 4.6. Statistical Analysis

Two-way analyses of variance (ANOVA) were performed on the differences between treatments and control conditions with SPSS 18.0 software. The statistics are displayed as means ± and standard errors (SE).

## 5. Conclusions

Based on the results obtained from this study, we can conclude that effects of CuO NPs significantly increased the morpho-physiological and biochemical traits of *B. juncea*. Such improvements can easily be observed in photosynthetic pigments and antioxidant defense systems. The response of seed-treated plants of 4 mg/L of CuO NPs (30 min) proved better than the other treatments. Overall, this study could provide a clear understanding for researchers to determine the actual molecular mechanism behind the CuO NPs-based enhancement mechanism in *B. juncea*, thereby enabling further investigation at the cellular level.

## Data Availability

Not applicable.
